# Analysis of Self-Care Activities in Type 2 Diabetes in Brazil: Protocol for a Scoping Review

**DOI:** 10.2196/49105

**Published:** 2024-03-20

**Authors:** Marileila Marques Toledo, Edson da Silva, Elizabethe Adriana Esteves

**Affiliations:** 1 Graduate Program in Health Sciences Universidade Federal dos Vales do Jequitinhonha e Mucuri (UFVJM) Diamantina Brazil; 2 Department of Basic Sciences Universidade Federal dos Vales do Jequitinhonha e Mucuri (UFVJM) Diamantina Brazil; 3 Graduate Program in Nutrition Sciences Universidade Federal dos Vales do Jequitinhonha e Mucuri (UFVJM) Diamantina Brazil

**Keywords:** type 2 diabetes mellitus, self-care, self-care activities, Brazil, diabetes mellitus, T2D, chronic disease, self-care activity, aging, ageing, methodological guidelines, knowledge, gaps, diabetes education, hyperglycemia, insulin resistance, health education

## Abstract

**Background:**

Diabetes mellitus is a chronic disease that is growing worldwide. It is estimated that 15.7 million people aged between 20 and 79 years live with diabetes in Brazil, and the majority of cases are type 2 diabetes (T2D). To successfully manage diabetes, the patient needs to develop self-care activities. However, there is limited understanding of what self-care activities are performed by people with T2D in Brazil.

**Objective:**

This study aims to identify and map studies that evaluate self-care activities in T2D in Brazil.

**Methods:**

This is a scoping review protocol structured according to the methodological guidelines of the Joanna Briggs Institute. Six databases and gray literature were used. The process of searching, identifying, and evaluating the papers was carried out by 2 independent reviewers, guided by the assumptions established by the Joanna Briggs Institute. We sought to answer the following guiding question: How are self-care activities for people with T2D evaluated in Brazil? We included papers and publications in any language, from public and private domains, and with different methodological approaches.

**Results:**

Initial database searches produced a total of 681 results. These papers will be critically analyzed, and relevant information will be extracted. Quantitative and qualitative results of the papers reviewed will be presented to respond to the study’s objective. We intend to publish the scoping review in the first half of 2024.

**Conclusions:**

The protocol for this scoping review will evaluate the main self-care activities carried out by adults and older people with T2D in Brazil. The results may help identify knowledge gaps and contribute to future research and diabetes education interventions.

**International Registered Report Identifier (IRRID):**

DERR1-10.2196/49105

## Introduction

Diabetes mellitus (DM) is a significant and growing health problem worldwide. In 2021, the International Diabetes Federation estimated that 8.8% (537 million) of the world’s population aged 20 to 79 years had diabetes. If current trends persist, the number of people with diabetes is projected to exceed 783 million by 2045. Brazil ranks sixth in the number of cases, with 15.7 million people diagnosed in the 20-79 years age group, which is projected to increase to 23.2 million cases in 2045 [1].

DM can be classified into 4 main types, based on its etiology: type 1 diabetes (T1D), type 2 diabetes (T2D), gestational DM, and other types of diabetes. T2D is the most prevalent form, accounting for approximately 90% to 95% of diabetes cases worldwide [2].

The causes of T2D are still poorly understood, but there is a strong link with excess weight, obesity, lack of physical activity, aging, ethnicity, and family history. T2D has an insidious onset and is characterized by insulin resistance, partial deficiency of insulin secretion by pancreatic β cells, and changes in incretin secretion [2,3].

There is evidence that T2D can be prevented or delayed [1]. However, the patient often remains undiagnosed with T2D for several years. T2D occurs when hyperglycemia is the result of insulin resistance that has been established gradually over many years until diagnosis [1,2]. In this case, the person does not present the classic signs of diabetes, such as dehydration or involuntary weight loss, and the diagnosis comes with acute or chronic complications of diabetes [2].

Diabetes complications are categorized as microvascular and macrovascular disorders. Thus, diabetes and its complications can lead to disability and constitute one of the main causes of early mortality in most countries [1,4].

For satisfactory management of diabetes and effective adjustment in lifestyle, it is necessary to develop self-care activities, which include adherence to a healthy diet, regular physical activity, blood glucose monitoring, foot care, medication use, and smoking cessation [5].

Self-care is defined as the ability of individuals, their families, and the community to promote and maintain their health, prevent disease, and manage their illness and disability with or without the support of a health professional [6].

The Association of Diabetes Care & Education Specialists (ADCES) emphasizes that diabetes self-care can be effective through 7 important behaviors (ADCES7 self-care behaviors [7]): (1) ensuring variety and balance between quality, quantity, and food safety (healthy eating)—fresh or minimally processed foods should be prioritized and thus form the basis of the diet, and ultraprocessed foods should be avoided; (2) engaging in physical activity and regular training with aerobic and resistance exercises (being active); (3) blood glucose monitoring, using reagent strips and glucometers or glucose monitoring with interstitial glucose sensors (controlling blood glucose values); (4) understanding and managing the use of medications at the correct times every day, according to medical prescription (taking medication); (5) being prepared to face unexpected complications, such as hyperglycemia or hypoglycemia, resulting from an error in carbohydrate counting, physical activity, use of alcoholic beverages, excess medication, or change in medication schedule (solving problems); (6) practicing behaviors that prevent or minimize chronic complications and adverse outcomes related to diabetes (reducing risks)—examples of these behaviors include making positive lifestyle changes and participating in a diabetes education program, and it is recommended to be up to date with laboratory and complementary tests (glycated hemoglobin, cholesterol levels, assessment of renal function, and fundus examination), clinical assessment, and emotional aspects; and (7) developing personalized strategies to face daily stress and know how to act in special situations such as travel, parties, and intercurrent illnesses (eg, infections and other clinical situations that impact blood glucose, etc). Seeking information and alternatives to adapt to different situations, thus benefiting glycemic control and self-efficacy, favors patients to face the challenges of diabetes (adapting healthily) [7].

There is vast scientific literature on diabetes and many self-care–related studies. However, to the best of our knowledge, no previous attempt to establish an analysis of different self-care activities in T2D focused on Brazilians. Thus, this protocol will guide a scoping review to identify and map studies evaluating self-care activities in T2D in Brazil. Scoping reviews are considered a type of literature review that maps and identifies the nature and extent of relevant evidence in a given field of research. It also seeks to identify gaps in evidence, clarify key concepts, and report on the types of evidence that address and inform practice in a thematic area [8].

This protocol will guide a scoping review to identify and map studies that evaluate self-care activities in T2D in Brazil.

## Methods

### Scoping Review

To maintain rigor, the scoping review methodology used for this protocol will be that of the *Joanna Briggs Institute (JBI) Reviewer’s Manual 2020* [9], which establishes five steps: (1) identification of research question; (2) identification of relevant studies; (3) selection of studies; (4) data analysis; and (5) grouping, synthesis, and presentation of data. The authors also followed the recommendations of the PRISMA-ScR (Preferred Reporting Items for Systematic Reviews and Meta-Analyses Extension for Scoping Reviews), according to the JBI Manual [9].

This study also followed all procedures standard to systematic reviews (search tests during elaboration of the protocol, data collection, data extraction, synthesis, analysis, and discussion of results and conclusions), as recommended by Cochrane (*Cochrane Handbook for Systematic Reviews of Interventions* [10]). A preliminary search of the PROSPERO Cochrane Database of Systematic Reviews and JBI Evidence Synthesis was performed. No existing or ongoing scoping reviews or systematic reviews were identified on this topic.

After completing the scoping review, the results will be submitted for publication in a peer-reviewed scientific journal. The research project for this scoping review protocol was previously registered in the Open Science Framework [11].

### Stage 1: Identifying the Research Question

The first step comprises the elaboration of a research question. This review followed the *Population*, *Concept*, and *Context* strategy for a scoping review [9]. The following were defined: people diagnosed with T2D (*Population*); actions or strategies to evaluate self-care, self-management, or self-efficacy of people with T2D (*Concept*); and studies developed in Brazil (*Context*). Thus, the guiding question of this study is as follows: How are self-care activities for people with T2D evaluated in Brazil?

### Stage 2: Identifying Relevant Studies

We performed a database search: (1) MEDLINE, (2) Web of Science - Core Collection, (3) Scopus, (4) Embase, (5) LILACS, and (6) SCIELO. In addition, a search was carried out for unpublished and possibly eligible studies in the gray literature, retrieved from 3 sources: the Brazilian Digital Library of Theses and Dissertations, Google Scholar (limited to the first 10 pages, in order of relevance), and the website of the Brazilian Diabetes Society. For the search strategy, terms were extracted from recognized thesauruses in the health area: Health Sciences Descriptors, Medical Subject Headings, and Embase Subject Headings. Thus, it started with the following terms: type 2 DM, self-management, self-efficacy, self-care, and Brazil. In order to broaden the search and better direct the findings, synonymous terms and the Boolean operators OR and AND were used according to the needs of each database (Multimedia Appendix 1).

As eligibility criteria, we included (1) Brazilian studies that were conducted with people diagnosed with T2D, older than 18 years, of both genders, and with or without comorbidities; (2) studies that focus on self-care activities for people with T2D; (3) studies that describe the evaluation process of these activities; and (4) studies that evaluate 2 or more self-care activities. The time frame used was the period from 2011 to 2023. The year 2011 is marked by the elaboration of the Strategic Action Plan for Combating Chronic Noncommunicable Diseases in Brazil 2011-2022 [12] and the Model of Attention to Chronic Conditions [13]. Original studies, dissertations, and theses were considered without language restriction, with quantitative, qualitative, or mixed design. Duplicate studies; studies on T1D, gestational diabetes, or prediabetes; studies that associate T2D with other types of diabetes; research protocols; editorials; reviews; letters; and Brazilian studies carried out in partnership with other countries were excluded.

### Stage 3: Study Selection

The identified literature was exported to the Mendeley reference manager (Elsevier), via the web version, to remove duplicates. It was then imported into the Rayyan (Qatar Computing Research Institute) systematic review management software, online version [14], for screening and selection of studies. The studies were selected independently by 2 previously trained researchers from the team, and a third researcher resolved conflicts in the absence of consensus. All identified studies’ titles and abstracts were evaluated based on the established inclusion and exclusion criteria. After the assessment of a study’s pertinence to the review question, it was selected for reading in full for later data extraction. Its references were analyzed in search of additional studies. This stage was developed based on the recommendations of the international guide PRISMA-ScR, with the presentation of a flowchart on the process of the search and selection of studies, quantitative results of each database, included or excluded studies, and the total number of works selected for evaluation and synthesis [15].

### Stage 4: Charting the Data

For the data extraction, a standardized data extraction tool was created in a Microsoft Office Excel Online spreadsheet, developed by the reviewers, and tested for this study in a pilot test based on the JBI tool [9] to characterize the studies. The instrument was independently used by 2 researchers to capture information to describe the extent and nature of the studies (authorship, year of publication, study typology, objectives primary results, and authors’ recommendations). Information was also collected, such as characteristics of the population, specific self-care activities (general food, specific food, physical activity, blood glucose monitoring, foot care, medication use, and smoking), and identification of diabetes self-care behaviors. If there was any disagreement between the data extracted by the 2 reviewers, it was resolved by dialogue between both reviewers or, if necessary, with a third reviewer. The studies were coded with letters and numbers following a logical sequence. Tables S1-3 in Multimedia Appendix 2 [7,16] are examples of the data extraction tools created for this study.

### Stage 5: Collating, Summarizing, and Reporting the Data

**Figure 1:**
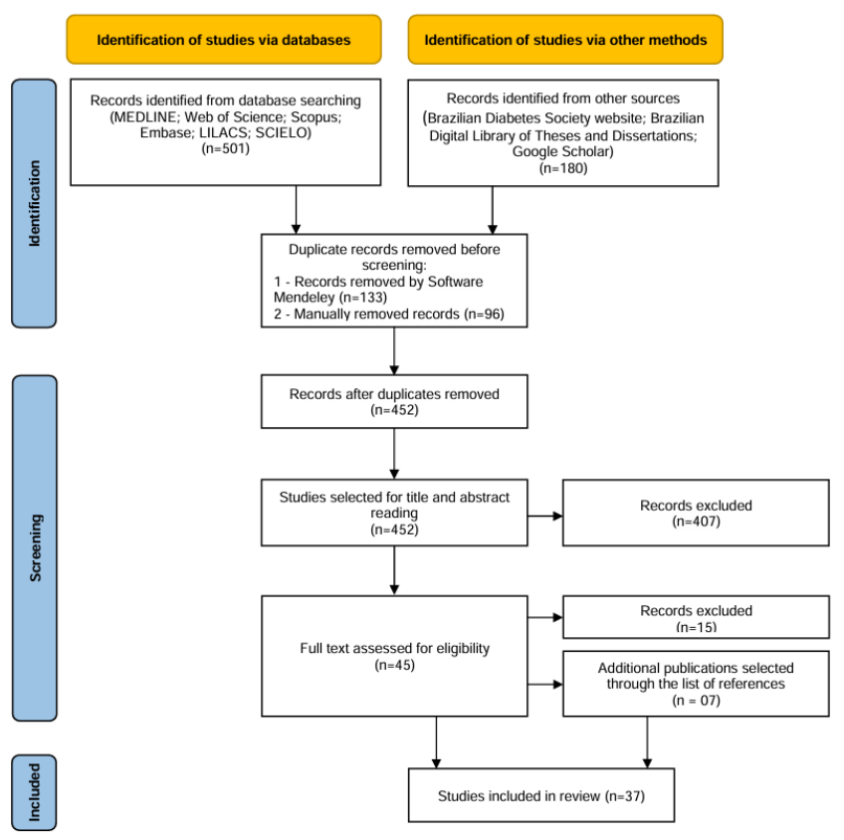
PRISMA (Preferred Reporting Items for Systematic Reviews and Meta-Analyses) flow diagram for the scoping review process.

Qualitative data will be analyzed using the Bardin content analysis technique [17] and descriptively by 2 authors and validated by the entire research team. As for quantitative data, Table S1 in Multimedia Appendix 2 will verify which self-care activities were carried out in each study and classify each activity as favorable or unfavorable. Table S3 in Multimedia Appendix 2 will verify which of the 7 self-care behaviors are addressed in the studies. The results will be presented in tables, graphs, and images, along with the construction of narratives to clarify the information and discuss it in light of relevant and updated literature. The scoping review will record and report reasons for excluding sources of evidence in the full text that do not meet the inclusion criteria. The review report will incorporate a PRISMA (Preferred Reporting Items for Systematic Reviews and Meta-Analyses) flow diagram (Figure 1), visually presenting the screening and selection process.

The assessment of methodological quality and risk of bias is optional [8] and will not be performed in our study.

### Ethical Considerations

This scoping review does not require ethical approval from the institutional review board.

## Results

In August 2023, our initial database searches identified 681 papers. Duplicates were removed, and screening of extracted titles and abstracts resulted in 37 papers. A critical evaluation analysis of the collected papers was carried out to extract relevant information. The screening was carried out in September 2023. Similarities and differences contained in various data from the papers are being analyzed and compared between September and November 2023. We estimate the publication of the scoping review will be in the first half of 2024.

## Discussion

### Principal Findings

Reviews that focus on self-care for adults and older people with T2D have been specific to educational interventions for self-care. To the best of our knowledge, this will be the first scoping review to understand how self-care activities carried out by people with T2D in Brazil are evaluated.

This scoping review will identify gaps in the literature and provide information on future research needs related to self-care assessment and its relationship to the 7 ADCES self-care behaviors [7] of adults and older adults with diabetes.

The method of this scoping review complies with the JBI recommendations [9] and the most recent guidelines for using the PRISMA-ScR tool [15], adapted for the study context. One of the benefits of using PRISMA is that it makes it possible to guide the writing of the research so that it reflects the investigative activities that will be carried out without losing details, in addition to guaranteeing methodological rigor [18]. Another advantage of a scoping review is the possibility of obtaining an overview of the scientific evidence about a given phenomenon in a structured, systematized, impartial, and transparent way [8].

The results of the scoping review will be discussed thoroughly based on the research question and will be discussed with the evidence reported in the literature on the research topic. We believe that our results will support the development of guidelines, thereby overcoming identified challenges and revealing new opportunities for assessing self-care behaviors in diabetes.

### Limitations

This scoping review will follow well-established methods, yet limitations will exist. First, most of the studies included are cross-sectional, which does not allow for long-term conclusions. Second, the noninclusion of texts published in other indexing databases and the choice to select studies only on the first 10 pages of Google Scholar may result in omitting potentially relevant research.

### Conclusions

This review will map and compare how the main self-care activities carried out by people with T2D in Brazil are evaluated. Our study will help identify existing knowledge gaps and how research on the topic is conducted. This knowledge will enable researchers and health care professionals to understand self-care activities in T2D better. Consequently, the results of our scoping review may contribute to future research and health education programs focusing on T2D.

## Appendix

Multimedia Appendix 1 Search strategy outlining concepts and alternative search terms.

Multimedia Appendix 2 Information about the following instruments: Data extraction instrument; Measurement of each self-care activity seven days a week; and Diabetes self-care behaviors.

## Abbreviations

ADCES: Association of Diabetes Care & Education Specialists
DM: diabetes mellitus
JBI: Joanna Briggs Institute
PRISMA: Preferred Reporting Items for Systematic Reviews and Meta-Analyses
PRISMA-ScR: Preferred Reporting Items for Systematic Reviews and Meta-Analyses Extension for Scoping Reviews
T1D: type 1 diabetes
T2D: type 2 diabetes

## Data Availability

The data sets analyzed during this study are available from the corresponding author on reasonable request.
