# Supplementary material for: Analysis of Self-Care Activities in Type 2 Diabetes in Brazil: Protocol for a Scoping Review
Source: JMIR Res Protoc. 2024 Mar 20;13:e49105. doi: 10.2196/49105 (PMC10993109; doi:10.2196/49105)
Supplement: Multimedia Appendix 1 [file resprot_v13i1e49105_app1.pdf]

Multimedia Appendix 1. Search strategy outlining concepts and alternate search terms. Diamantina, MG, Brasil. 2024.

Data base	Strategy
MEDLINE	((("diabetes mellitus, type 2"[MeSH Terms]) OR ("diabetes mellitus, type 2" OR "type 2 diabetes mellitus" OR "diabetes mellitus, type II" OR "diabetes mellitus, type 2" OR "type 2 diabetes" OR "diabetes, type 2" OR "diabetes mellitus, non insulin dependent" OR "diabetes mellitus, non-insulin-dependent" OR "diabetes mellitus, noninsulin dependent" OR "diabetes mellitus, noninsulin-dependent" OR "non-insulin-dependent diabetes mellitus" OR "noninsulin dependent diabetes mellitus" OR "noninsulin-dependent diabetes mellitus" OR "NIDDM" OR "adult-onset diabetes mellitus" OR "diabetes mellitus, adult onset" OR "diabetes mellitus, adult-onset" OR "diabetes mellitus, slow onset" OR "diabetes mellitus, slow-onset" OR "diabetes mellitus, maturity onset" OR "diabetes mellitus, maturity-onset" OR "maturity onset diabetes" OR "maturity onset diabetes mellitus" OR "maturity-onset diabetes" OR "maturity-onset diabetes mellitus" OR "diabetes, maturity-onset" OR "MODY" OR "ketosis-resistant diabetes mellitus" OR "stable diabetes mellitus" OR "diabetes mellitus, stable")) AND (((("self care"[MeSH Terms]) OR ("self care" OR "self care" OR "care, self" OR "self-care")) OR (("self-management"[MeSH Terms]) OR ("self-management" OR "self management"))) OR (("self efficacy"[MeSH Terms]) OR ("self efficacy" OR "efficacy, self")))) AND ((brazil*[MeSH Terms]) OR (brazil*))
WEB OF SCIENCE	((TS=("Type 2 Diabetes Mellitus" OR "Diabetes Mellitus, Type II" OR "Diabetes Mellitus, Type 2" OR "Diabetes Mellitus, Non Insulin Dependent" OR "Diabetes Mellitus, Non-Insulin-Dependent" OR "Diabetes Mellitus, Noninsulin Dependent" OR "Diabetes Mellitus, Noninsulin-Dependent" OR "Non-Insulin-Dependent Diabetes Mellitus" OR "Noninsulin Dependent Diabetes Mellitus" OR "Noninsulin-Dependent Diabetes Mellitus" OR niddm OR "Type 2 Diabetes" OR "Diabetes, Type 2" OR "Adult-Onset Diabetes Mellitus" OR "Diabetes Mellitus, Adult Onset" OR "Diabetes Mellitus, Adult-Onset" OR "Diabetes Mellitus, Slow Onset" OR "Diabetes Mellitus, Slow-Onset" OR "Diabetes Mellitus, Maturity Onset" "Diabetes Mellitus, Maturity-Onset" OR "Maturity Onset Diabetes" OR "Maturity Onset Diabetes Mellitus" OR "Maturity-Onset Diabetes" OR "Maturity-Onset Diabetes Mellitus" OR "Diabetes, Maturity-Onset" OR mody OR "Ketosis-Resistant Diabetes Mellitus" OR "Stable Diabetes Mellitus")) AND TS=("Care, Self" OR "Self-Care" OR "Self-Management" OR "Self Management" OR "Self Efficacy")) AND TS=(Brazil*)
SCOPUS	(TITLE-ABS-KEY ("type 2 diabetes mellitus" OR "diabetes mellitus, type II" OR "diabetes mellitus, type 2" OR "type 2 diabetes" OR "diabetes, type 2" OR "diabetes mellitus, non insulin dependent" OR "diabetes mellitus, non-insulin-dependent" OR "diabetes mellitus, noninsulin dependent" OR "diabetes mellitus, noninsulin-dependent" OR "non-insulin-dependent diabetes mellitus" OR "noninsulin dependent diabetes mellitus" OR "noninsulin-dependent diabetes

	mellitus" OR "NIDDM" OR "adult-onset diabetes mellitus" OR "diabetes mellitus, adult onset" OR "diabetes mellitus, adult-onset" OR "diabetes mellitus, slow onset" OR "diabetes mellitus, slow-onset" OR "slow-onset diabetes mellitus" OR "diabetes mellitus, maturity onset" OR "diabetes mellitus, maturity-onset" OR "maturity onset diabetes" OR "maturity onset diabetes mellitus" OR "maturity-onset diabetes" OR "maturity-onset diabetes mellitus" OR "diabetes, maturity-onset" OR "MODY" OR "ketosis-resistant diabetes mellitus" OR "diabetes mellitus, ketosis resistant" OR "diabetes mellitus, ketosis-resistant" OR "stable diabetes mellitus" OR "diabetes mellitus, stable") AND TITLE-ABS-KEY ("self care" OR "care, self" OR "self-care" OR "self-management" OR "self management" OR "self efficacy") AND TITLE-ABS-KEY (brazil*))
EMBASE	#1 AND #2 AND #3<br>#1<br>('non insulin dependent diabetes mellitus'/exp OR 'adult onset diabetes':ti,ab,kw OR 'adult onset diabetes mellitus':ti,ab,kw OR 'diabetes mellitus type 2':ti,ab,kw OR 'diabetes mellitus type ii':ti,ab,kw OR 'diabetes mellitus, maturity onset':ti,ab,kw OR 'diabetes mellitus, non insulin dependent':ti,ab,kw OR 'diabetes mellitus, non-insulin-dependent':ti,ab,kw OR 'diabetes mellitus, type 2':ti,ab,kw OR 'diabetes mellitus, type ii':ti,ab,kw OR 'diabetes type 2':ti,ab,kw OR 'diabetes type ii':ti,ab,kw OR 'diabetes, adult onset':ti,ab,kw OR 'dm 2':ti,ab,kw OR 'insulin independent diabetes':ti,ab,kw OR 'insulin independent diabetes mellitus':ti,ab,kw OR 'ketosis resistant diabetes mellitus':ti,ab,kw OR 'maturity onset diabetes':ti,ab,kw OR 'maturity onset diabetes mellitus':ti,ab,kw OR 'niddm':ti,ab,kw OR niddm:ti,ab,kw OR 'non insulin dependent diabetes mellitus':ti,ab,kw OR 'non insulin dependent diabetes':ti,ab,kw OR 'non-insulin-dependent diabetes mellitus':ti,ab,kw OR 'noninsulin dependent diabetes':ti,ab,kw OR 'noninsulin dependent diabetes mellitus':ti,ab,kw OR 't2dm':ti,ab,kw OR 'type 2 diabetes':ti,ab,kw OR 'type 2 diabetes mellitus':ti,ab,kw OR 'type ii diabetes':ti,ab,kw OR 'type ii diabetes mellitus':ti,ab,kw) AND [embase]/lim<br>#2<br>('self care'/exp OR 'concept, self'/exp OR 'self care':ti,ab,kw OR 'self management':ti,ab,kw OR 'self treatment':ti,ab,kw OR 'self-management':ti,ab,kw OR 'self-nurturance':ti,ab,kw OR 'selfcare':ti,ab,kw OR 'selfmanagement':ti,ab,kw OR 'selftreatment':ti,ab,kw OR 'concept, self':ti,ab,kw OR 'self':ti,ab,kw OR 'self awareness':ti,ab,kw OR 'self confrontation':ti,ab,kw OR 'self efficacy':ti,ab,kw OR 'self image':ti,ab,kw OR 'self perception':ti,ab,kw OR 'self rating':ti,ab,kw OR 'self representation':ti,ab,kw OR 'selfconcept':ti,ab,kw OR 'self concept':ti,ab,kw) AND [embase]/lim<br>#3<br>('brazilian'/exp OR 'brazil'/exp OR brazilian:ti,ab,kw OR brazilians:ti,ab,kw OR brazil:ti,ab,kw OR 'federative republic of brazil':ti,ab,kw OR 'united states of brazil':ti,ab,kw) AND [embase]/lim

LILACS	("Diabetes Mellitus Tipo 2" OR "Diabetes Mellitus Tipo II" OR "Diabetes Tipo 2" OR "Diabetes Mellitus não Insulinodependente" OR "Diabetes Mellitus não Dependente de Insulina" OR dmnid OR "Diabetes Mellitus de Início no Adulto" OR "Diabetes Mellitus de Início Gradativo" OR mody OR "Diabetes Mellitus de Início na Maturidade" OR "Diabetes Mellitus Resistente a Cetose" OR "Diabetes Mellitus Estável" OR "Type 2 Diabetes Mellitus" OR "Diabetes Mellitus, Type II" OR "Diabetes Mellitus, Type 2" OR "Diabetes Mellitus, Non Insulin Dependent" OR "Diabetes Mellitus, Non-Insulin-Dependent" OR "Diabetes Mellitus, Noninsulin Dependent" OR "Diabetes Mellitus, Noninsulin-Dependent" OR "Non-Insulin-Dependent Diabetes Mellitus" OR "Noninsulin Dependent Diabetes Mellitus" OR "Noninsulin-Dependent Diabetes Mellitus" OR niddm OR "Type 2 Diabetes" OR "Diabetes, Type 2" OR "Adult-Onset Diabetes Mellitus" OR "Diabetes Mellitus, Adult Onset" OR "Diabetes Mellitus, Adult-Onset" OR "Diabetes Mellitus de Inicio Adulto" OR "Diabetes Mellitus, Slow Onset" OR "Diabetes Mellitus, Slow-Onset" OR "Slow-Onset Diabetes Mellitus" OR "Diabetes Mellitus, Maturity Onset" "Diabetes Mellitus, Maturity-Onset" OR "Maturity Onset Diabetes" OR "Maturity Onset Diabetes Mellitus" OR "Maturity-Onset Diabetes" OR "Maturity-Onset Diabetes Mellitus" OR "Diabetes, Maturity-Onset" OR mody OR "Ketosis-Resistant Diabetes Mellitus" OR "Stable Diabetes Mellitus" OR "Diabetes Mellitus no Insulinodependiente" OR "Diabetes Mellitus no Insulino-Dependiente" OR "Diabetes Mellitus de Inicio en la Madurez" OR "Diabetes Mellitus de Inicio Lento" OR "Diabetes Mellitus de Inicio Adulto" OR "Diabetes Mellitus Resistente a la Cetosis" OR "Diabetes Mellitus Estable" OR dmim) AND (autocuidado OR autoajuda OR autogestão OR autogerenciamento OR "Auto-Gestão" OR "Auto Gestão" OR "Auto Gerenciamento" OR autoeficácia OR "Self Care" OR "Care, Self" OR "Self-Care" OR "Self-Management" OR "Self Management" OR "Self Efficacy" OR autoayuda OR automanejo OR autoeficacia) AND (brasil* OR brazil*) AND (db:("LILACS"))
SciELO	("diabetes mellitus tipo 2" OR "type 2 diabetes mellitus" OR "diabetes mellitus, type 2" OR "diabetes tipo 2" OR "diabetes do tipo 2" OR "type 2 diabetes" OR "diabetes, type 2" OR "diabetes mellitus tipo II" OR "diabetes mellitus, type II" OR "diabetes mellitus não insulinodependente" OR "diabetes mellitus não dependente de insulina" OR DMNID OR "diabetes mellitus, non insulin dependent" OR "diabetes mellitus, non-insulin-dependent" OR "diabetes mellitus, noninsulin dependent" OR "diabetes mellitus, noninsulin-dependent" OR "non-insulin-dependent diabetes mellitus" OR "noninsulin dependent diabetes mellitus" OR "noninsulin-dependent diabetes mellitus" OR NIDDM OR "diabetes mellitus no insulinodependiente" OR "diabetes mellitus no insulino-dependiente" OR DMNID OR "diabetes mellitus de início gradativo" OR "diabetes mellitus, slow onset" OR "diabetes mellitus, slow-onset" OR "slow-onset diabetes mellitus" OR "diabetes mellitus de inicio lento" OR "diabetes mellitus de início no adulto" OR "adult-onset diabetes mellitus" OR "diabetes mellitus, adult onset" OR "diabetes mellitus, adult-onset" OR "diabetes mellitus de inicio adulto" OR "diabetes mellitus de inicio adulto" OR MODY OR "diabetes mellitus de

	início na maturidade" OR "diabetes mellitus, maturity onset" OR "diabetes mellitus, maturity-onset" OR "maturity onset diabetes" OR "maturity onset diabetes mellitus" OR "maturity-onset diabetes" OR "maturity-onset diabetes mellitus" OR "diabetes, maturity-onset" OR "diabetes mellitus de inicio en la madurez" OR DMIM OR "diabetes mellitus resistente a cetose" OR "ketosis-resistant diabetes mellitus" OR "diabetes mellitus, ketosis resistant" OR "diabetes mellitus, ketosis-resistant" OR "diabetes mellitus resistente a la cetosis" OR "diabetes mellitus estável" OR "stable diabetes mellitus" OR "diabetes mellitus, stable" OR "diabetes mellitus estable") AND (autocuidado OR "self care" OR "care, self" OR "self-care" OR autoajuda OR autogestão OR "auto-gestão" OR "auto gestão" OR autogerenciamento OR "auto gerenciamento" OR "auto-gerenciamento" OR "self-management" OR "self management" OR autoayuda OR automanejo OR autoeficácia OR "self efficacy" OR autoeficacia) AND (brasil* OR brazil*)
BDTD	(Todos os campos:"diabetes mellitus tipo 2" E Todos os campos: autocuidado OR autogestão OR autoeficácia E Todos os campos: brasil*)
Google Scholar	("diabetes mellitus tipo 2" OR "diabetes mellitus, type 2") AND (autocuidado OR "self care" OR autoayuda OR autogestão OR "self-management" OR automanejo OR autoeficácia OR "self efficacy" OR autoeficacia) AND (brasil* OR brazil*)
Website of the Brazilian Diabetes Society.	autocuidado

Source: authors.
