# Supplementary material for: Analysis of Self-Care Activities in Type 2 Diabetes in Brazil: Protocol for a Scoping Review
Source: JMIR Res Protoc. 2024 Mar 20;13:e49105. doi: 10.2196/49105 (PMC10993109; doi:10.2196/49105)
Supplement: Multimedia Appendix 2 [file resprot_v13i1e49105_app2.pdf]

Table S1. Data extraction instrument. Diamantina, MG, Brazil. 2024.

**Reviewer: ______________________________**	**Extraction date: __/__/__**			
Items to be extracted	Study 1	Study 2	Study 3	...
1- Citation (authors, title, periodical, year of publication)				
2 - Country of origin/Brazilian state/context*				
3- Goals				
4 - Study design				
5 - Characteristics of participants (>18 years old; type 2 diabetes mellitus)	☐Yes<br>☐No	☐Yes<br>☐No	☐Yes<br>☐No	☐Yes<br>☐No
6 - Data collection instrument used in the study				
7 - Do you evaluate more than one self-care domain**?	☐Yes<br>☐No	☐Yes<br>☐No	☐Yes<br>☐No	☐Yes<br>☐No
8 - According to the ADCES7 Self-Care Behaviors™ framework, which diabetes self-care behaviors are addressed***?	☐1<br>☐2<br>☐3<br>☐4<br>☐5<br>☐6<br>☐7	☐1<br>☐2<br>☐3<br>☐4<br>☐5<br>☐6<br>☐7	☐1<br>☐2<br>☐3<br>☐4<br>☐5<br>☐6<br>☐7	☐1<br>☐2<br>☐3<br>☐4<br>☐5<br>☐6<br>☐7
9 - Main results				
10 - Conclusions/Recommendations/Implications for practice described by the authors				
11- Additional studies				

*Context: Place where the study was carried out and other relevant details (urban, rural). If the setting is not adequately described, use the name of the city/town/geographical location.
**Domains: General food; Specific food; Physical activity; Blood glucose monitoring; Foot care; Use of medication; Smoking.
***ADCES7 Self-Care Behaviors™: 1 - Healthy coping; 2 - Healthy eating; 3 - Being active; 4 - Taking medication; 5 - Monitoring; 6 - Problem solving; 7 - Reducing risks.

Source: Adapted from [1].

Table S2. Measurement of each self-care activity for seven days a week. Diamantina, MG, Brazil. 2024.

**Study code**	**Self-care domains**																
	**General food**		**Specific food**			**Physical activity**		**Blood glucose monitoring**		**Foot care**			**Medication use**			**Smoking**	
	Followed a healthy diet	Followed dietary guidance given by a health professional	Ate five or more servings of fruits and/or vegetables	Ate foods rich in fat	Ingested sweets	Performed physical activity for at least 30 minutes	Performed specific physical exercise	Assessed blood sugar	Assessed blood sugar the number of times recommended by a professional	Examined the feet	Examined your shoes before putting them on	Dried your feet between your toes after washing them	Took medications as recommended	Took insulin injections as recommended	Took the indicated number of diabetes pills	No	Yes
Study 1																	
Study 2																	
Study 3																	
….																	

Measurement of each self-care activity: >=5 days a week= favorable; <5 days a week= not favorable; unrated item=0; smoking: no=favorable; yes=no favorable; unrated item=0. Reverse scoring for items 4 and 5: >=5 days a week= not favorable; <5 days a week= favorable; unrated item=0;

Source: Adapted from [16].

Table S3. Diabetes self-care behaviors. Diamantina, MG, Brazil. 2024.

**Study code**	**Diabetes Self-care behaviors**						
	1- Healthy coping	2 - Healthy eating	3 - Being active	4 - Taking medication	5 - Monitoring	6 - Reducing risks	7 - Problem solving
Study 1							
Study 2							
Study 3							
…							

Source: Adapted from [7].
